# Coffee and the risk of osteoarthritis: a two-sample, two-step multivariable Mendelian randomization study

**DOI:** 10.3389/fgene.2024.1340044

**Published:** 2024-02-01

**Authors:** Wenzheng Zhang, Xuefeng Lei, Yihui Tu, Tong Ma, Tao Wen, Tao Yang, Long Xue, Jiazhong Ji, Huaming Xue

**Affiliations:** Department of Orthopaedics, Yangpu Hospital, School of Medicine, Tongji University, Shanghai, China

**Keywords:** osteoarthritis, coffee type, coffee intake, Mendelian randomization, mediating effects

## Abstract

**Purpose:** To investigate the potential causal relationship between coffee consumption and osteoarthritis (OA), and to disentangle whether body mass index (BMI) and Bone mineral density (BMD) mediate this relationship.

**Methods:** We performed two-sample and two-step Mendelian randomization (MR) analyses utilizing publicly available genome-wide association studies (GWAS) summary statistics to estimate the association between coffee intake and OA risk (including knee OA, hip OA, knee or hip OA, and total OA), as well as the possible mediating effects of BMI and BMD. In addition, data of different coffee types (decaffeinated coffee, instant coffee, ground coffee—including espresso, filter, etc., and other coffee types) were used to explore the effect of coffee type on the risk of OA.

**Results:** In two-sample MR, coffee intake increased the risk of OA in various sites, with the most significant impact observed in knee osteoarthritis (KOA) (odds ratio [OR] 2.03, 95% confidence interval [CI] 1.57–2.61, *p* < 0.001). The effect on self-reported OA was minimal (OR 1.03, 95% CI 1.01–1.05, *p* = 0.006). Further analysis of different types of coffee revealed that only decaffeinated coffee was causally associated with both KOA (OR 4.40, 95% CI 1.71–11.33, *p* = 0.002) and self-reported OA (OR 1.13, 95% CI 1.02–1.26, *p* = 0.022). In two-step MR, BMI explained over half of the coffee intake-all OA risk association, while BMD accounted for less than 5% of the mediation effect.

**Conclusion:** Our study suggests that coffee intake increase the risk of OA, with BMI playing a significant mediating role. Decaffeinated coffee appears to have the greatest impact on OA risk compared to other types of coffee. Therefore, managing BMI and selecting appropriate types of coffee should be included in the health management of individuals who frequently consume coffee.

## 1 Introduction

Osteoarthritis (OA) is a common chronic degenerative disease that affects the knee, hip, and hand joints, leading to chronic pain, stiffness, and movement disorders (Martel-Pelletier et al., 2016; Zhang et al., 2023). The pathogenesis of OA is complex and can be influenced by various factors such as poor lifestyle habits and health status, including excessive physical labor (Wang et al., 2020), body mass index (BMI) (Ho et al., 2022), overweight and obesity (Reyes et al., 2016). Previous studies have reported conflicting results on the relationship between coffee consumption and OA risk (Bang et al., 2019; Lim et al., 2022). This highlights the importance of determining whether coffee intake has a causal impact on the risk of OA, especially in the context of the European League Against Rheumatology (EULAR) emphasis on the role of lifestyle in skeletal muscle diseases (Gwinnutt et al., 2023).

Coffee is one of the most popular beverage consumed worldwide and has been the subject of extensive research regarding its impact on various health conditions including cardiovascular diseases, diabetes, cancers, and mental and neurological disorders (Butt and Sultan, 2011; Alicandro et al., 2017). The complex composition of coffee contains numerous active substances that can have diverse effects on health (Terentes-Printzios and Vlachopoulos, 2022). For instance, caffeic acid, commonly found in coffee, has been shown to inhibit inflammatory reactions and may have a protective effect on OA (Huang et al., 2018). Furthermore, the flavonoids present in coffee exhibit anti-inflammatory and antioxidant properties, which could potentially be therapeutically beneficial for regulating metabolism and the progression of inflammatory bone diseases (Ortiz et al., 2022).

However, it is important to recognize that coffee as a whole cannot be equated to any single component. Moreover, there are various types of coffee available in the market, each with its own unique composition. Previous studies have shown an association between coffee and BMI (Lee et al., 2019) as well as Bone mineral density (BMD) (Tański et al., 2021). Therefore, it is worth exploring whether BMI and BMD mediate the relationship between coffee intake and OA. Observational studies are susceptible to confounding factors and reverse causality, which can lead to biased results. The emergence of Mendelian randomization provides a novel approach to address these limitations (Zhang et al., 2023).

Mendelian randomization, as a methodology employing genetic variation as an instrumental variable, serves to evaluate the causal association between exposure and outcomes. In recent years, it has been widely used in the exploration of causative factors and potential targets of OA, as well as interactions between OA and a wide range of other diseases due to its ability to mitigate the effects of confounders and avoid reverse causality bias (Zhang et al., 2022; Fu et al., 2023). Although previous Mendelian studies have suggested a potential causal association between coffee intake and OA risk (Lee, 2018; Zhong et al., 2019; Zhang et al., 2021), such conclusions should be interpreted with caution due to a limited number of single nucleotide polymorphisms (SNPs) used, low statistical power, and potential confounding factors. Additionally, there is no research yet on whether the causal relationship between different types of coffee and OA risk differs.

In this study, our primary objectives are to elucidate the causal association between various types of coffee consumption and the risk of OA. Additionally, we aim to investigate the potential mediating roles of BMI and BMD in the relationship between coffee intake and OA.

## 2 Materials and methods

### 2.1 Study design and data source

**FIGURE 1:**
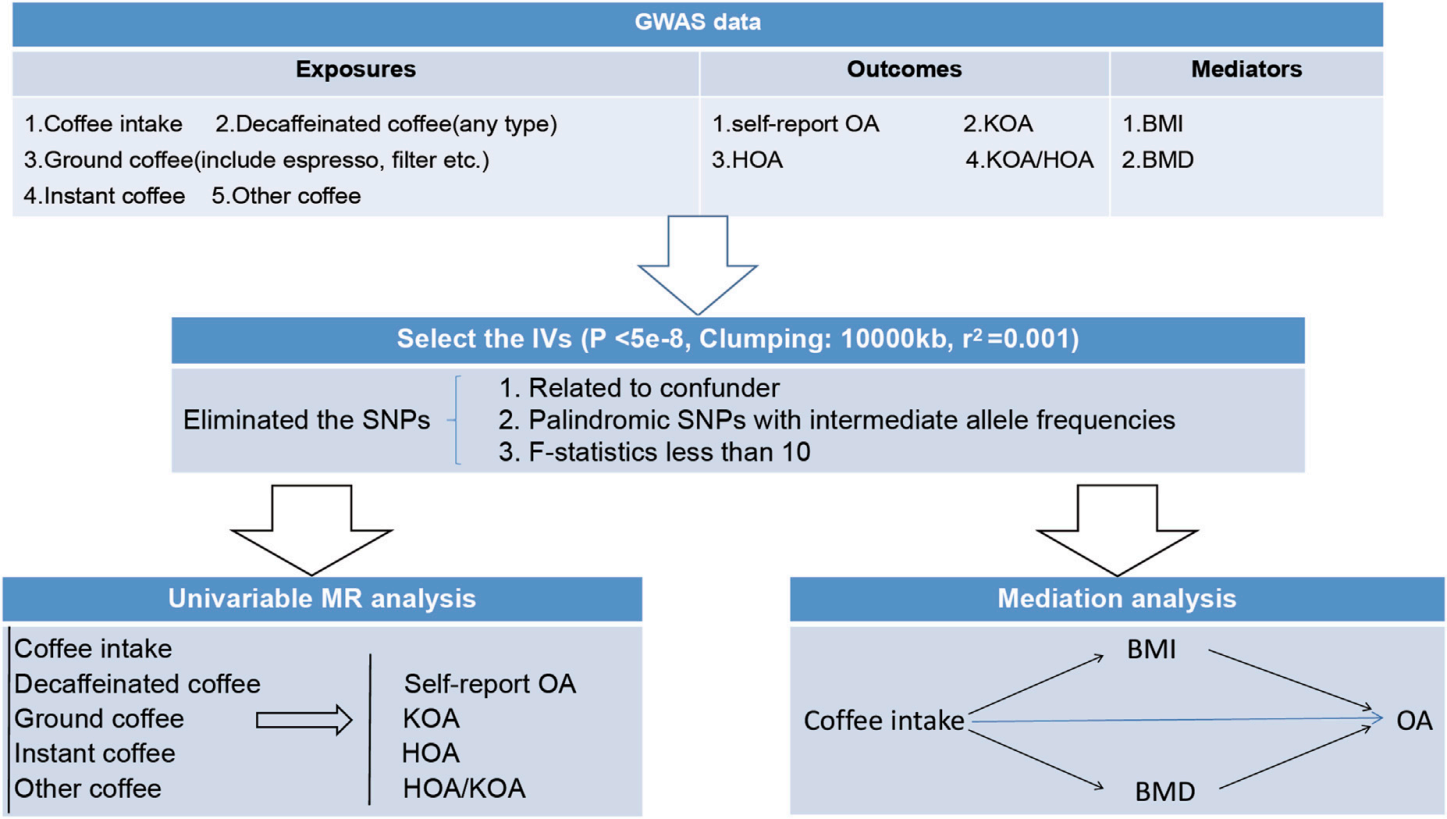
The overview design for the present study. OA, osteoarthritis. KOA, knee osteoarthritis. HOA, hip osteoarthritis. BMI, body mass index. BMD, Bone mineral density, SNPs, single nucleotide polymorphisms.

As shown in Figure 1, we commenced our study by conducting a univariate Mendelian randomization analysis. This analysis enabled us to examine the potential existence of a causative relationship between diverse forms of coffee consumption and OA. Moreover, we fortified our outcomes by incorporating external validation through the utilization of OA data extracted from the comprehensive research (Zengini et al., 2018). Subsequently, we employed two-step and two-sample Mendelian randomization analyses utilizing data on coffee intake, BMI, and BMD from relevant GWAS. These analyses sought to ascertain the potential mediating effects of BMI and BMD in the association between coffee intake and OA. Overall, our research endeavor follows a meticulous design, encompassing comprehensive assessments of distinct coffee intake types, external validation, and the examination of mediating factors such as BMI and BMD. By adopting this approach, we aim to contribute valuable insights to the existing literature on the intricate relationship involving coffee consumption, BMI, BMD, and the risk of OA.

SNPs associated with coffee intake and different coffee type choices were extracted from GWAS of more than 320,000 UKB participants. These individuals were queried about their daily coffee intake, as well as their typical choice of coffee type (decaffeinated coffee (any type), instant coffee, ground coffee - including espresso, filter, etc., and other coffee types) (Canela-Xandri et al., 2018), released by Neale lab (http://www.nealelab.is/uk-biobank/). Genetic associations with BMI with 461,460 participants were obtained from the GWAS summary data (https://gwas.mrcieu.ac.uk/). Summary-level statistic for BMD with 365,403 participants were downloaded from the GEnetic Factors for OSteoporosis Consortium website (GEFOS, http://www.gefos.org/). The primary outcome of this study was osteoarthritis (OA) and its various subtypes, which encompassed self-reported OA, knee OA (KOA), hip OA (HOA), and knee or hip OA (462,933/403,124/393,873/417,596 participants, respectively). And the data of self-reported OA used in this study were extracted from the GWAS summary data (https://gwas.mrcieu.ac.uk/). The remaining OA data were obtained from genomewide meta-analysis for OA (Tachmazidou et al., 2019). In addition to the data mentioned earlier, summary-level statistics from the study conducted by Zengini et al. (2018) were utilized for external validation purposes (63,556/22,347/11,989/32,970 participants, respectively). All participants were limited to individuals of European descent, which helped to mitigate potential biases resulting from population stratification. Detailed description of the characteristics of the GWAS related to the exposures, mediator, and outcomes can be found in Supplementary Table S1. As all the data used in this study were publicly accessible, ethical approval was not required for this research.

### 2.2 Selection of instrumental variables

To be considered a valid instrumental variable (IV) estimator, the following three assumptions must be met: 1) it should be strongly and consistently associated with the exposure (relevance condition); 2) it should be independent of the outcome (exclusion restriction condition); 3) it should be unrelated to any potential confounding variables with respect to both the exposures and outcomes (exchange ability condition) (Burgess et al., 2017). We extracted SNPs robustly associated with exposure (P < 5e-8, or relaxed to P < 5e-6). From this set, only independent SNPs (kb = 10,000, r2 < 0.001) were retained based on the linkage disequilibrium (LD) structure of European populations, and the palindromic SNPs with intermediate allele frequencies were excluded. In addition, the phenoscanner tool was employed to ascertain whether any of the selected instrument SNPs were associated with confounding factors. To mitigate weak instrumental variable bias, we calculated the F-statistic, which was calculated using the following equation: F = R2 (SampleSize-2)/(1-R2) (Palmer et al., 2012). We applied a filter to exclude weak instruments with an F-statistic lower than 10.

### 2.3 Univariate MR analysis

We used the inverse variance weighted (IVW) as the main MR analysis, which utilizes a random effects model meta-analysis method to combine the outcomes from a single SNP and achieve a more precise estimate of the causal connection between the exposures and outcomes (Chen et al., 2023). The estimates were derived from a random effects model due to the *p*-value < 0.05 for heterogeneity based on Cochran’s Q statistic, otherwise, the estimates were derived from a fixed-effects MR estimates model. Furthermore, we utilized both the MR Egger and weighted median methods to investigate the relationship between coffee intake and OA. Additionally, we evaluated the potential presence of horizontal pleiotropy through the MR-Egger intercept and the MR-PRESSO global test. The MR-PRESSO outlier test was utilized to detect any possible outliers (Verbanck et al., 2018). In case an outlier SNP was identified (*p* < 0.05), we recalculated the causal effects by excluding the outliers and employing the remaining SNPs.

### 2.4 Mediation analysis

For mediation analysis, we conducted a two-step MR. In the first step, we evaluate the causal relationship between exposure and mediation variables (beta1). Subsequently, in the second step, we estimated the causal effect of mediators on outcomes through multivariate MR (beta2). We then calculated the total effect (beta0) between exposure and outcome using two-sample MR analysis. When beta0, beta1, and beta2 were all significant, a causal relationship existed between the outcome and exposure, and the mediating variable played a partial mediational role in this causal relationship. The mediating effect was calculated using beta1 * beta2, while the mediating proportion of the causal effect between exposure and outcome was calculated using beta1 * beta2/beta0 (Chen et al., 2022). Finally, we estimated the proportion of the mediation effect in the total effect using the delta method (Carter et al., 2019). We calculated the odds ratios (OR) and 95% confidence interval (CI) to measure causal effects.

We conducted causal estimates using the “TwoSampleMR” (Hemani et al., 2018) package and detected outliers using the “MR-PRESSO” package. For MVMR analysis, we utilized the “MVMR” and “Mendelian Randomization” (Yavorska and Burgess, 2017) packages. We performed all statistical analyses using R software version 4.3.2.

## 3 Results

### 3.1 Univariate MR analysis

**FIGURE 2:**
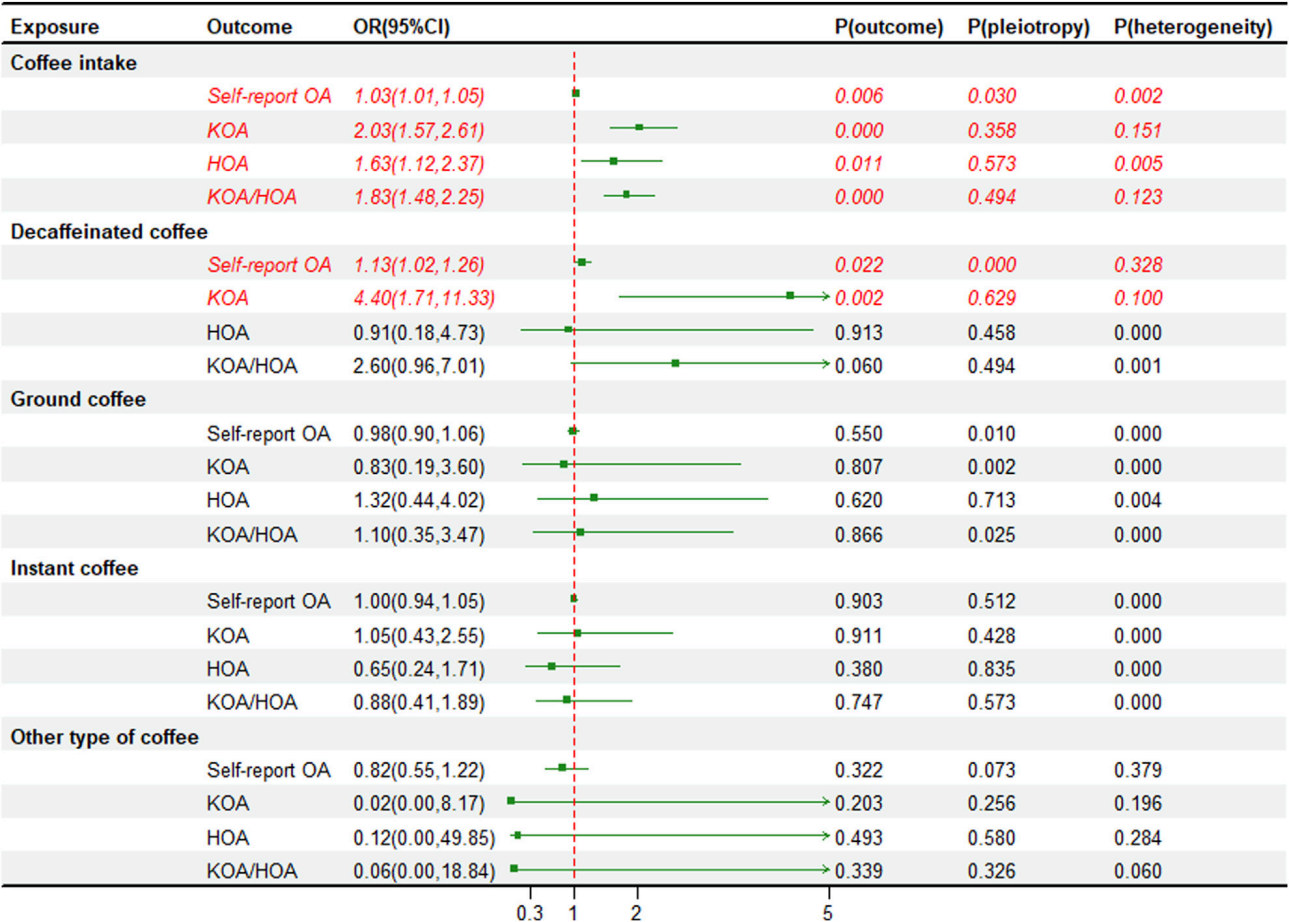
Forest plot of the main analysis of coffee intake and osteoarthritis risk. OA, osteoarthritis; KOA, knee osteoarthritis; HOA, hip osteoarthritis; OR, odds ratios; CI, confidence interval; *p*-value for pleiotropy test of univariable MR Egger. *p*-value for heterogeneity based on Cochran’s Q statistic.

**FIGURE 3:**
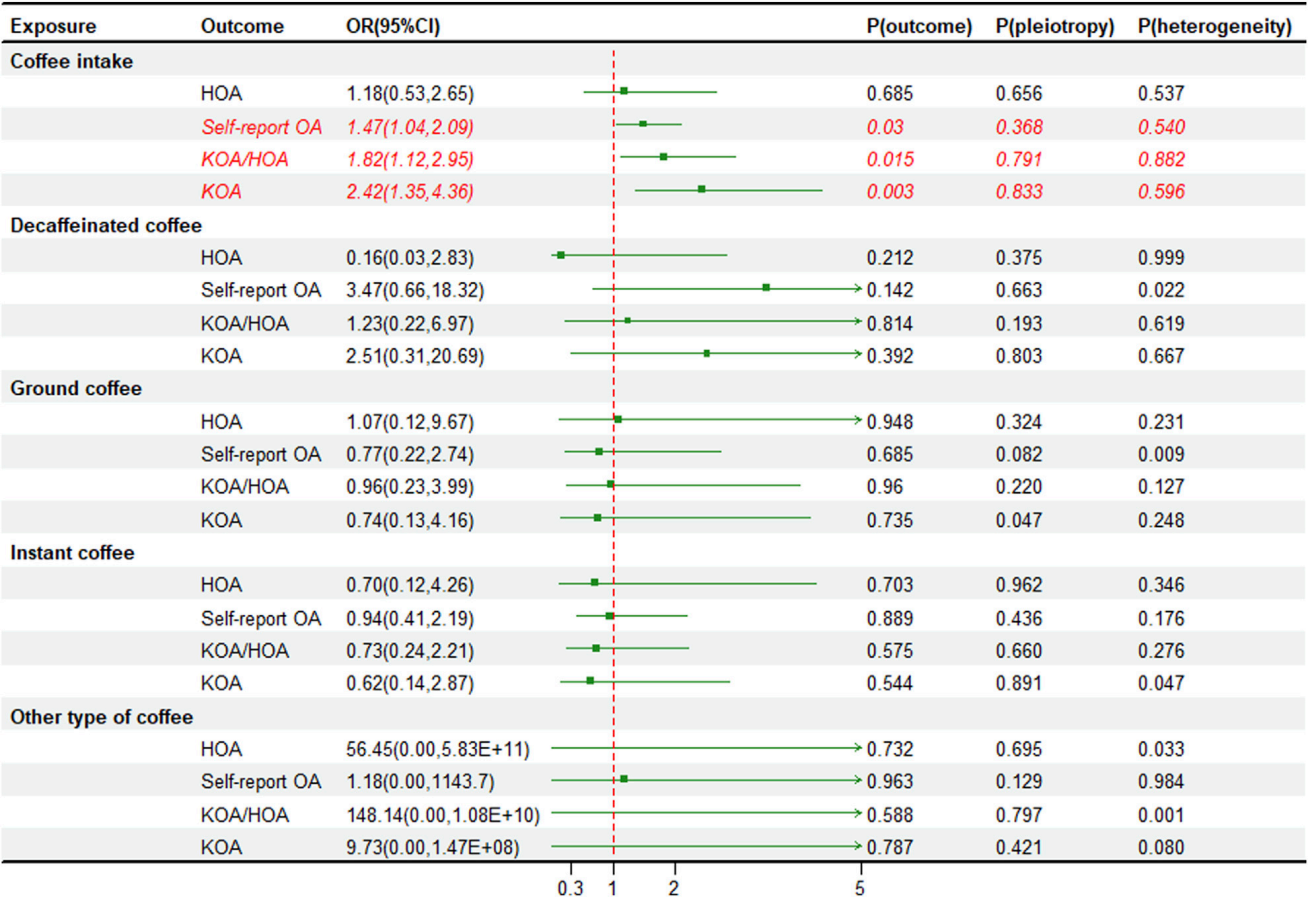
External validation forest plot of coffee intake and osteoarthritis risk. OA, osteoarthritis; KOA, knee osteoarthritis; HOA, hip osteoarthritis; OR, odds ratios; CI, confidence interval; *p*-value for pleiotropy test of univariable MR Egger. *p*-value for heterogeneity based on Cochran’s Q statistic.

We utilized instrumental variables (SNPs) that were significantly correlated with the exposure for two sample MR. In addition, we excluded SNPs that showed strong correlations with BMI, type 2 diabetes, and OA, then calculated the F-statistical for the remaining SNPs (Supplementary Tables S2–S6). The univariate MR analysis indicates a causal relationship between coffee intake and the risk of OA in various parts, with the strongest impact observed in KOA (OR 2.03, 95% CI 1.57–2.61, *p* < 0.001) and minimal impact on self-reported OA (OR 1.03, 95% CI 1.01–1.05, *p* = 0.006). Interestingly, we found that only decaffeinated coffee had a significant association with KOA (OR 4.40, 95% CI 1.71–11.33, *p* = 0.002) and self-reported OA (OR 1.13, 95% CI 1.02–1.26, *p* = 0.022) (Figure 2). We also performed external validation and found a consistent causal relationship between coffee intake and OA in various parts, although there was no statistically significant correlation between coffee intake and HOA (OR 1.18, 95% CI 0.53–2.65, *p* = 0.685). Furthermore, after classifying coffee, no causal relationship was observed between classified coffee and any part of OA (Figure 3). Similar results were shown in the MR methods weighted median and MR-Egger (Supplementary Tables S7–S11). The individual effect estimates of SNPs exhibited some heterogeneity, although no outliers were identified by MR-PRESSO. Based on the results of the MR-Egger intercept test, our analysis indicated a minimal presence of horizontal pleiotropy (Figures 2, 3). Additionally, the “Leave-one-out plot” revealed that none of the SNPs had a dominant effect on the estimated causal association between coffee intake and OA (Supplementary Figures S1–S4).

### 3.2 Mediating effect of BMI between coffee intake and OA

We utilized a two-step MR to perform the mediation analysis. The results revealed a significant causal association between gene-predicted coffee intake and BMI (beta1 = 0.724, *p* = 4.44E-05). In addition, multivariate Mendelian analysis illustrated that BMI significantly impacted all types of OA, including self-reported OA, KOA, HOA, and HOA/KOA (beta2 = 0.036, *p* = 4.49E-65; beta2 = 0.808, *p* = 1.79E-106; beta2 = 0.483, *p* = 1.99E-29; beta2 = 0.668, *p* = 1.55E-112) (Table 1). The impact of coffee on OA was analyzed, and the results indicated significant effects on all types of OA (Figure 1). The significant values of Beta0, Beta1, and Beta2 demonstrate a causal relationship between coffee intake and OA, with BMI partially mediating this relationship. Specifically, the final causal effects mediating self-reported OA, KOA, HOA, and KOA/HOA were found to be 71.11%, 67.90%, 45.12%, and 60.56%, respectively. Furthermore, indirect effects were estimated using the detal method, further strengthens our conclusion.

**TABLE 1:** The two-step Mendelian randomization estimates for mediator body mass index between coffee intake and osteoarthritis.

Exposure	Outcome	Method	nsnp	Beta	SE	Pval	Midbeta	Mid_of_sum	*p* (detal)
Coffee intake	BMI	IVW	39	0.724	0.177	4.44E-05	0.026	0.711	7.17E-05
BMI	self-reported OA	IVW	440	0.036	0.002	4.49E-65			
Coffee intake	self-reported OA	IVW	39	0.037	0.010	1.30E-04			
Coffee intake	BMI	IVW	39	0.724	0.177	4.44E-05	0.585	0.679	5.97E-05
BMI	KOA	IVW	441	0.808	0.037	1.79E-106			
Coffee intake	KOA	IVW	39	0.862	0.132	6.43E-11			
Coffee intake	BMI	IVW	39	0.724	0.177	4.44E-05	0.349	0.451	1.24E-04
BMI	HOA	IVW	439	0.483	0.043	1.99E-29			
Coffee intake	HOA	IVW	39	0.774	0.204	1.48E-04			
Coffee intake	BMI	IVW	39	0.724	0.177	4.44E-05	0.483	0.606	5.87E-05
BMI	KOA/HOA	IVW	441	0.668	0.030	1.55E-112			
Coffee intake	KOA/HOA	IVW	39	0.798	0.125	1.95E-10			

BMI, body mass index. IVW, inverse-variance weighted. SE, standard deviation. Midbeta, mediating effect from exposure to the outcome. Mid_of_sum, the proportion of the mediating effect within the causal relationship.

The estimates were derived from a random effects model due to the *p*-value<0.05 for heterogeneity based on Cochran’s Q statistic, otherwise, The estimates were derived from a fixed-effects MR, estimates model.

### 3.3 Mediating effect of BMD between coffee intake and OA

As shown in Table 2, we found a causal relationship between coffee intake and BMD (beta1 = 0.159, *p* = 0.024). In addition, BMD had a significant impact on OA in various sites, including self-reported OA, KOA, HOA, and HOA/KOA (beta2 = 0.003, *p* = 0.033; beta2 = 0.091, *p* = 0.001; beta2 = 0.113, *p* = 0.001; beta2 = 0.095, *p* = 2.89E-05). The significance of Beta0, Beta1, and Beta2 suggests the presence of a causal associations between coffee intake and OA, with BMD acting as a partial mediator. The final causal effects mediating self-reported OA, KOA, HOA, and KOA/HOA were estimated to be 1.48%, 1.69%, 2.33%, and 1.89%, respectively. However, when we calculated the standard error of indirect effects using the detal method, we found that BMD was only statistically significant in the case of KOA/HOA (*p* = 0.047).

**TABLE 2:** The two-step Mendelian randomization estimates for mediator Bone mineral density between coffee intake and osteoarthritis.

Exposure	Outcome	Method	nsnp	Beta	SE	Pval	Midbeta	Mid_of_sum	*p* (detal)
Coffee intake	BMD	IVW	35	0.159	0.071	0.024	0.001	0.015	0.111
BMD	self-reported OA	IVW	413	0.004	0.002	0.033			
Coffee intake	self-reported OA	IVW	39	0.037	0.010	0.000			
Coffee intake	BMD	IVW	35	0.159	0.071	0.024	0.015	0.017	0.060
BMD	KOA	IVW	440	0.091	0.027	0.001			
Coffee intake	KOA	IVW	39	0.862	0.132	0.000			
Coffee intake	BMD	IVW	35	0.159	0.071	0.024	0.018	0.023	0.061
BMD	HOA	IVW	439	0.113	0.034	0.001			
Coffee intake	HOA	IVW	39	0.774	0.204	0.000			
Coffee intake	BMD	IVW	35	0.159	0.071	0.024	0.015	0.019	0.047
BMD	KOA/HOA	IVW	439	0.095	0.023	0.000			
Coffee intake	KOA/HOA	IVW	39	0.798	0.125	0.000			

BMD, bone mineral density; IVW, inverse-variance weighted; SE, standard deviation; Midbeta, mediating effect from exposure to the outcome. Mid_of_sum, the proportion of the mediating effect within the causal relationship.

The estimates were derived from a random effects model due to the *p*-value<0.05 for heterogeneity based on Cochran’s Q statistic, otherwise, The estimates were derived from a fixed-effects MR, estimates model.

## 4 Discussion

OA is a leading cause of disability among the elderly population. Since there are limited effective treatment options available, it is essential to reduce the risk of OA through lifestyle modifications. Coffee is one of the most widely consumed beverages worldwide and has been extensively studied for its potential health effects. Although some previous studies have suggested an increased risk of OA associated with coffee intake (Lee, 2018; Zhong et al., 2019; Zhang et al., 2021), it is important to note that coffee contains certain compounds, such as caffeic acids and flavonoids, which possess anti-inflammatory properties. These compounds could potentially act as protective factors against the development and progression of OA (Huang et al., 2018; Terentes-Printzios and Vlachopoulos, 2022). To further investigate the relationship between coffee consumption and the risk of OA, we conducted a comprehensive analysis using GWAS data with a larger sample size. Additionally, we carried out external validation to reinforce our conclusions. In line with previous studies, our research indicates that coffee consumption may indeed be associated with an increased risk of OA. Furthermore, we conducted a preliminary exploration of the potential pathways through which coffee intake might elevate the risk of OA. Utilizing a two-step MR analysis, we determined that BMI and BMD mediate the causal relationship between coffee intake and OA risk. It is important to emphasize that while our study provides valuable insights into the association between coffee consumption and OA risk, further research is needed to comprehend the underlying mechanisms in greater detail. Additionally, investigating the specific impact of different types of coffee on OA risk is also warranted. Nevertheless, our findings contribute to the expanding literature in this field and underscore the potential role of coffee consumption in the development of OA.

The composition of coffee is very complex and includes hundreds of different substances, of which the most familiar component is caffeine (Bang et al., 2019; Lim et al., 2022). There is numerous data suggesting that caffeine intake is associated with a high risk of osteoarthritis. In rat models, it has been observed that exposure of maternal rats to low-doses of caffeine affects the joint integrity of their foetuses (Tan et al., 2012; Tan et al., 2018), which may be due to the antagonistic effect of caffeine on adenosine receptors as well as increased osteoclastogenesis (Yi et al., 2016; Nieber, 2017). Interestingly, however, some studies have reported an inhibitory effect of caffeine on certain pro-inflammatory cytokines, such as interleukin (IL)-1 and tumor necrosis factor alpha (TNF-α) (Ullah et al., 2015). Furthermore, coffee comprises a variety of polyphenolic compounds, including chlorogenic acids and caffeic acids, known for their antioxidant and anti-inflammatory properties (Heinecke et al., 2010). A study found that intra-articular injections of chlorogenic acid not only altered the expression of inflammatory mediators, but also reduced cartilage degradation in experimental osteoarthritis (Chen et al., 2011). In addition, coffee contains a variety of flavonoids, such as quercetin, neohesperidin and hesperidin, which have a variety of biological activities (Xue et al., 2019; Ortiz et al., 2022). *In vitro* studies have shown that these flavonoids have anti-osteoclastogenic and anti-inflammatory effects. They decrease the levels of reactive oxygen species and matrix metalloproteinases and inhibit the expression of pro-inflammatory cytokines and osteoclast markers (Ortiz et al., 2022). Although past studies have revealed an association between caffeine and an increased risk of osteoarthritis, almost all of the other coffee components that have been studied have anti-inflammatory effects and restrain the development of osteoarthritis, our study found that consumption of decaffeinated coffee increased the risk of osteoarthritis. Therefore, further research is still needed to find out exactly what chemical components are responsible for the increased risk of osteoarthritis. In addition, different roasting and brewing methods and types of coffee also affect the chemical composition of coffee. By further exploring the relationship between the composition of coffee and osteoarthritis, we can better understand the effects of coffee on human health.

Importantly, there are various types of coffee available in the market, each with its own unique combination of ingredients. For instance, roasted coffee contains specific polyphenols (chlorogenic acidsCGAs and melanins) which possess antioxidant properties (Scalbert and Williamson, 2000; Yanagimoto et al., 2004). In addition, roasted coffee contains polyphenols and melanoids that play a significant role as antioxidants in diet. A study of 83 commercially available coffee varieties found that unmixed and ground coffee had the highest CGAs content, while having the lowest average caffeine/CGAs ratio (Jeon et al., 2019). However, focusing solely on coffee intake as an exposure factor may not be clinically significant. Therefore, in our study, we examined different types of coffee to better understand their impact on OA risk. We conducted two-sample MR using four types of coffee (decaffeinated coffee, ground coffee, instant coffee, and other types of coffee) as exposures, and the results indicated that only decaffeinated coffee increased the risk of self-reported OA and KOA, rather than all types of coffee exhibiting this effect. Furthermore, there was a significant difference in the upper and lower limits of the 95% CI for the results of other types of coffee. This may be due to the imprecise classification of other coffee types, including coffee types that have a completely opposite impact on OA. Overall, our findings suggest that it is advisable to minimize the consumption of decaffeinated coffee in order to reduce the risk of OA. However, it is important to acknowledge that the data utilized in this study primarily originated from the European population and may be susceptible to demographic cross-biases. Additionally, some types of coffee showed horizontal pleiotropy, indicating that further research in this area using larger samples and richer cohorts is warranted. Furthermore, a more precise classification of coffee types is necessary to minimize intra-group differences. Steps could also be taken to mitigate the potential harm of coffee consumption by reducing harmful ingredients. Therefore, it is important to carefully evaluate these results and take them into consideration when making decisions about coffee consumption.

BMI is widely recognized as an important risk factor in the occurrence and development of OA (Bouchard et al., 2010; Moran-Lev et al., 2023). Indeed, coffee has a complex composition and contains CGA, which has been associated with reducing the BMI of overweight individuals (Watanabe et al., 2019). Additionally, caffeine itself has been found to be beneficial for weight control (Kulkarni et al., 2016). However, the inclusion of artificial flavoring agents in coffee to enhance its taste has been associated with a higher BMI (Lee and Kean, 2012). It is important to note that there is still no definitive conclusion on whether coffee intake directly leads to an increase in BMI, as multiple confounding factors can influence this relationship. In our study, we conducted a two-step mediation analysis to investigate the causal relationship between coffee intake and OA risk, with BMI acting as a mediator. The results showed that coffee intake does lead to an increase in BMI, and further analysis demonstrated that BMI plays a mediating role in the relationship between coffee intake and OA risk, with a mediation ratio exceeding 50%. This suggests that closely monitoring BMI and implementing measures to control it through diet or exercise can effectively reduce the risk of OA among individuals who consume coffee frequently. It is important to consider these findings and take appropriate steps to maintain a healthy BMI in order to mitigate the risk of developing OA, especially for individuals who regularly consume coffee.

Previous studies have identified a notable association between coffee intake and 12 blood metabolites, with three of these metabolites (AFMU, 3-hydroxyhippuric acid, and triglyceride) impacting BMD (Chau et al., 2020). Furthermore, Hartley et al. (2022) reported that BMD directly affects the occurrence of OA, independent of BMI. Hence, we postulate that BMD might serve as a mediating factor in the relationship between coffee consumption and OA risk. To investigate this hypothesis, we conducted a two-step MR imaging analysis and confirmed the presence of a mediating effect exerted by BMD. However, when employing the detal method to calculate the indirect effects, we observed statistical significance (*p* = 0.0471) only in relation to the influence of coffee intake on KOA/HOA. Moreover, the mediating ratios were consistently below 5%. Consequently, the practical clinical significance of BMD as a mediator in the causal association between coffee and OA may be limited.

This study is subject to several limitations that need to be acknowledged. Firstly, all the GWAS data utilized in this study were derived exclusively from the European population, thus warranting caution when extrapolating the findings to other populations. Secondly, our pleiotropy analysis revealed the presence of pleiotropic effects in certain coffee varieties on OA, which partially impacts the stability of the results. Additionally, despite employing methods such as MR Egger and MR PROSSO to control for confounding factors, it is need to recognize that residual confounding factors may still exist. Lastly, our investigation solely focused on exploring the mediating effects of BMI and BMD, while neglecting various other potential factors that remain unexplored.

## 5 Conclusion

This study reaffirms that coffee intake is associated with an increased risk of OA, particularly highlighting the elevated risks associated with decaffeinated coffee intake in terms of KOA and self-reported OA. Additionally, we identified BMI as a significant mediator in the causal relationship between coffee consumption and OA, whereas the mediating role of BMD appears to be relatively minor. Consequently, monitoring and managing BMI levels may potentially reduce the risk of OA among coffee consumers. Furthermore, when selecting coffee types, reducing the selection of decaffeinated variants may be preferable to mitigate OA risk.

## Data Availability

The original contributions presented in the study are included in the article/Supplementary Material, further inquiries can be directed to the corresponding author.
