# Supplementary material for: Coffee and the risk of osteoarthritis: a two-sample, two-step multivariable Mendelian randomization study
Source: Front Genet. 2024 Feb 1;15:1340044. doi: 10.3389/fgene.2024.1340044 (PMC10867243; doi:10.3389/fgene.2024.1340044)
Supplement: Supplementary file 1 [file Table1.DOCX]

Supplementary Material

# Supplementary Data

Supplementary Table 1 Data Source

Supplementary Table 2 Genetic instruments of Decaffeinated coffee( any type) for Two-sample MR analysis.

Supplementary Table 3 Genetic instruments of Ground coffee (include espresso, filter etc) for Two-sample MR analysis.

Supplementary Table 4 Genetic instruments of Instant coffee for Two-sample MR analysis.

Supplementary Table 5 Genetic instruments of Other type of coffee for Two-sample MR analysis.

Supplementary Table 6 Genetic instruments of coffee intake for Two-sample MR analysis.

Supplementary Table 7 MR analysis for coffee intake and OA in various sites.

Supplementary Table 8 MR analysis for Decaffeinated coffee and OA in various sites.

Supplementary Table 9 MR analysis for Ground coffee and OA in various sites.

Supplementary Table 10 MR analysis for Instant coffee and OA in various sites.

Supplementary Table 11 MR analysis for Other type of coffee and OA in various sites.

Supplementary figure 1 MR Leave-one-out sensitivity analysis for Decaffeinated coffee and OA in various sites.

Supplementary figure 2 MR Leave-one-out sensitivity analysis for Ground coffee and OA in various sites.

Supplementary figure 3 MR Leave-one-out sensitivity analysis for Instant coffee and OA in various sites.

Supplementary figure 4 Supplementary figure 4 MR Leave-one-out sensitivity analysis for Other coffee and OA in various sites.

**Supplementary Table 1 Data Source**

| **Phenotype** | **GWAS ID** | **Sample size** | **Publication** | **Link** |
| --- | --- | --- | --- | --- |
| Coffee intake /  Choice for different coffee types | [ukb-b-5237](https://gwas.mrcieu.ac.uk/datasets/ukb-b-5237/)  [ukb-d-1508](https://gwas.mrcieu.ac.uk/datasets/ukb-d-1508_1/) | 389,665/ 329,671 | Nealelab | http://www.nealelab.is/uk-biobank/ |
| BMI | [ukb-b-19953](https://gwas.mrcieu.ac.uk/datasets/ukb-b-19953/) | 461,460 | MRC-IEU | https://gwas.mrcieu.ac.uk/ |
| BMD | ebi-a-GCST90014022 | 407,746 | Computationally efficient whole-genome regression for quantitative and binary traits | https://pubmed.ncbi.nlm.nih.gov/34017140/ |
| Self-reported OA for main analysis | [ukb-b-14486](https://gwas.mrcieu.ac.uk/datasets/ukb-b-14486/) | 462,933 | MRC-IEU | https://gwas.mrcieu.ac.uk/ |
| KOA/HOA/KOA or HOA for main analysis | [ebi-a-GCST007090](https://gwas.mrcieu.ac.uk/datasets/ebi-a-GCST007092/)  [ebi-a-GCST007091](https://gwas.mrcieu.ac.uk/datasets/ebi-a-GCST007092/)  [ebi-a-GCST007092](https://gwas.mrcieu.ac.uk/datasets/ebi-a-GCST007092/) | 403,124/393,873/417,596 | Identification of new therapeutic targets for osteoarthritis through genome-wide analyses of UK Biobank data | https://pubmed.ncbi.nlm.nih.gov/30664745/ |
| Self-reported OA/ KOA/ HOA/ HOA or KOA for external validation | [ebi-a-GCST005810](https://gwas.mrcieu.ac.uk/datasets/ebi-a-GCST005811/)  [ebi-a-GCST005811](https://gwas.mrcieu.ac.uk/datasets/ebi-a-GCST005811/)  [ebi-a-GCST005812](https://gwas.mrcieu.ac.uk/datasets/ebi-a-GCST005811/)  [ebi-a-GCST005813](https://gwas.mrcieu.ac.uk/datasets/ebi-a-GCST005811/) | 63,556/22,347/11,989/32,970 | Genome-wide analyses using UK Biobank data provide insights into the genetic architecture of osteoarthritis | https://pubmed.ncbi.nlm.nih.gov/29559693/ |

**Supplementary Table 2 Genetic instruments of Decaffeinated coffee( any type) for MR analysis**

| **SNP** | **chr** | **pos** | **effect_allele** | **other_allele** | **beta** | **se** | **pval** | **F.statistics** |
| --- | --- | --- | --- | --- | --- | --- | --- | --- |
| rs7532549 | 1 | 93539656 | T | G | -0.0053226 | 0.0011117 | 1.69E-06 | 22.9245481 |
| rs12615894 | 2 | 52313952 | C | T | -0.0072226 | 0.0015292 | 2.32E-06 | 22.3080334 |
| rs116237319 | 2 | 145241652 | C | T | -0.014843 | 0.0029452 | 4.66E-07 | 25.3995246 |
| rs13107325 | 4 | 103188709 | T | C | 0.0160937 | 0.0019959 | 7.46E-16 | 65.0141869 |
| rs112308424 | 4 | 37679792 | C | G | -0.0123185 | 0.0023714 | 2.05E-07 | 26.9846635 |
| rs112764911 | 4 | 7527405 | C | A | -0.0120174 | 0.0022967 | 1.67E-07 | 27.3793945 |
| rs28472599 | 5 | 66514625 | G | A | 0.0116232 | 0.0025369 | 4.61E-06 | 20.9922225 |
| rs116266375 | 5 | 109704751 | C | A | -0.0301309 | 0.0064592 | 3.09E-06 | 21.7603935 |
| rs164839 | 5 | 106988605 | A | T | -0.0071602 | 0.0014001 | 3.15E-07 | 26.1539355 |
| rs11966489 | 6 | 157414029 | C | T | 0.0060559 | 0.0012796 | 2.22E-06 | 22.3990342 |
| rs145736159 | 6 | 158861039 | T | G | -0.0191433 | 0.0034672 | 3.37E-08 | 30.4844474 |
| rs7857828 | 9 | 135046069 | A | G | 0.00651949 | 0.0013832 | 2.44E-06 | 22.2152398 |
| rs17496153 | 9 | 6070037 | C | G | 0.00845432 | 0.0016332 | 2.26E-07 | 26.7968620 |
| rs55969935 | 11 | 66646365 | T | C | -0.0090139 | 0.0018633 | 1.31E-06 | 23.4032827 |
| rs61902817 | 11 | 112915157 | G | A | -0.0073962 | 0.0015265 | 1.26E-06 | 23.4779593 |
| rs7189450 | 16 | 50389838 | G | A | -0.0089221 | 0.0019368 | 4.09E-06 | 21.2207974 |
| rs57603836 | 17 | 13115504 | G | T | 0.0125328 | 0.0027129 | 3.84E-06 | 21.3424688 |
| rs34504554 | 19 | 54550558 | G | T | -0.0049451 | 0.0010763 | 4.34E-06 | 21.1083359 |

_*Removing the SNP “rs13107325” for being related to BMI._

**Supplementary Table 3 Genetic instruments of Ground coffee (include espresso, filter etc) for Two-sample MR analysis**

| **SNP** | **chr** | **pos** | **effect_allele** | **other_allele** | **beta** | **se** | **pval** | **F.statistics** |
| --- | --- | --- | --- | --- | --- | --- | --- | --- |
| rs324416 | 1 | 46865387 | G | A | -0.00899128 | 0.00147887 | 1.20E-09 | 36.96434769 |
| rs66495454 | 1 | 72748567 | GTCCT | G | 0.00846063 | 0.00115469 | 2.36E-13 | 53.68767495 |
| rs309137 | 2 | 136765951 | C | T | 0.0079237 | 0.0013847 | 1.05E-08 | 32.74497508 |
| rs975924 | 2 | 59166256 | C | T | 0.00626374 | 0.00112294 | 2.44E-08 | 31.11389206 |
| rs70943009 | 2 | 10090764 | AT | A | 0.00638745 | 0.00113376 | 1.76E-08 | 31.74042706 |
| rs62168767 | 2 | 77332740 | A | C | 0.00858535 | 0.00153958 | 2.46E-08 | 31.09649838 |
| rs7572185 | 2 | 12629763 | A | G | -0.0100241 | 0.00167807 | 2.32E-09 | 35.68376203 |
| rs7613360 | 3 | 49916710 | T | C | -0.00808754 | 0.00113546 | 1.06E-12 | 50.73284347 |
| rs58799235 | 5 | 59855103 | C | T | 0.00642039 | 0.00112793 | 1.26E-08 | 32.40100816 |
| rs7742854 | 6 | 98528143 | T | C | 0.00720348 | 0.00113955 | 2.60E-10 | 39.95930615 |
| rs989134 | 6 | 26336224 | C | A | 0.0063428 | 0.00111665 | 1.35E-08 | 32.26471955 |
| rs2106727 | 7 | 17287998 | G | A | 0.00706755 | 0.00116068 | 1.14E-09 | 37.07769923 |
| rs11782665 | 8 | 14262647 | C | A | -0.00663354 | 0.00113964 | 5.87E-09 | 33.88093106 |
| rs112701574 | 10 | 99053230 | CT | C | 0.00766107 | 0.00134202 | 1.14E-08 | 32.58823841 |
| rs7897379 | 10 | 65301725 | C | T | 0.00689555 | 0.00111405 | 6.04E-10 | 38.3114384 |
| rs7948839 | 11 | 66634397 | T | C | -0.00681274 | 0.00123021 | 3.06E-08 | 30.66797665 |
| rs12323725 | 14 | 30145211 | G | A | -0.0062832 | 0.00114594 | 4.19E-08 | 30.06339407 |
| rs2472297 | 15 | 75027880 | T | C | 0.0111042 | 0.00125194 | 7.38E-19 | 78.66970463 |
| rs9938265 | 16 | 9905841 | G | A | 0.00669464 | 0.00122723 | 4.90E-08 | 29.75791711 |
| rs369989480 | 16 | 28836378 | TA | T | -0.0094987 | 0.00117336 | 5.74E-16 | 65.53386542 |
| rs16958414 | 16 | 82772171 | G | T | -0.00746279 | 0.00135614 | 3.74E-08 | 30.28261604 |
| rs75014231 | 18 | 21072433 | C | T | -0.00760448 | 0.00116515 | 6.74E-11 | 42.59664211 |

_*Removing the following SNPs for being related to BMI:rs7613360, rs7742854._

**Supplementary Table 4 Genetic instruments of Instant coffee for Two-sample MR analysis**

| **SNP** | **chr** | **pos** | **effect_allele** | **other_allele** | **beta** | **se** | **pval** | **F.statistics** |
| --- | --- | --- | --- | --- | --- | --- | --- | --- |
| rs834233 | 1 | 150354276 | A | G | 0.00632104 | 0.00136667 | 3.74E-06 | 21.39191945 |
| rs1192831 | 1 | 247455719 | A | G | -0.00800757 | 0.00157627 | 3.78E-07 | 25.80716316 |
| rs3789044 | 1 | 204589101 | A | G | -0.00896323 | 0.00159248 | 1.82E-08 | 31.67970348 |
| rs61783464 | 1 | 9335339 | T | C | -0.00694668 | 0.00139686 | 6.59E-07 | 24.731407 |
| rs13400338 | 2 | 125857102 | C | A | -0.00693314 | 0.00133266 | 1.97E-07 | 27.0658216 |
| rs6736768 | 2 | 59176413 | G | A | -0.00813909 | 0.00153047 | 1.05E-07 | 28.28147523 |
| rs11690828 | 2 | 77326548 | A | T | -0.00863685 | 0.00175329 | 8.39E-07 | 24.26628213 |
| rs201145892 | 2 | 212754754 | T | C | -0.00690794 | 0.0015106 | 4.81E-06 | 20.91212436 |
| rs1014174 | 2 | 229951964 | C | T | 0.00739866 | 0.00132574 | 2.40E-08 | 31.14507782 |
| rs75734647 | 3 | 179250765 | C | T | -0.016377 | 0.00304388 | 7.44E-08 | 28.94767205 |
| rs6810182 | 3 | 129062351 | T | C | -0.00652049 | 0.00133922 | 1.12E-06 | 23.70590903 |
| rs114831445 | 4 | 15919114 | A | G | -0.0268469 | 0.00530765 | 4.24E-07 | 25.58490077 |
| rs72803914 | 5 | 141709629 | A | T | 0.0289057 | 0.00626403 | 3.94E-06 | 21.29410171 |
| rs6892116 | 5 | 17356543 | C | T | 0.00623587 | 0.00134056 | 3.29E-06 | 21.638223 |
| rs9402795 | 6 | 136549346 | T | C | 0.0130101 | 0.00284835 | 4.94E-06 | 20.86289452 |
| rs7779393 | 7 | 4811174 | A | G | -0.0164399 | 0.00339549 | 1.29E-06 | 23.4419336 |
| rs4410790 | 7 | 17284577 | C | T | -0.00661514 | 0.00136931 | 1.36E-06 | 23.33858492 |
| rs1440755 | 8 | 57437149 | T | C | -0.00701837 | 0.00152705 | 4.31E-06 | 21.12350614 |
| rs56391050 | 8 | 40234378 | C | T | 0.00805335 | 0.00173667 | 3.53E-06 | 21.50396496 |
| rs3827811 | 8 | 8659038 | T | C | 0.00673781 | 0.00144231 | 2.99E-06 | 21.82329337 |
| rs7034200 | 9 | 4289050 | A | C | -0.00620254 | 0.0013158 | 2.43E-06 | 22.22078428 |
| rs76462340 | 9 | 27009291 | G | C | 0.018902 | 0.00391552 | 1.38E-06 | 23.30433016 |
| rs10989557 | 9 | 104349215 | T | C | 0.00628585 | 0.00132865 | 2.24E-06 | 22.38240978 |
| rs11191266 | 10 | 104108414 | A | G | 0.0167411 | 0.00361413 | 3.62E-06 | 21.45657708 |
| rs2393986 | 10 | 65320006 | T | A | -0.00718027 | 0.00131618 | 4.89E-08 | 29.76123686 |
| rs1291818 | 10 | 11132190 | C | T | 0.0062005 | 0.00133852 | 3.62E-06 | 21.45871413 |
| rs77458514 | 10 | 130410033 | T | C | -0.00974465 | 0.00195244 | 6.01E-07 | 24.91019327 |
| rs35107470 | 15 | 74817689 | G | A | -0.00828385 | 0.00143985 | 8.76E-09 | 33.10014918 |
| rs2352749 | 16 | 10192778 | A | T | -0.00907768 | 0.00181663 | 5.83E-07 | 24.96989837 |
| rs2466826 | 16 | 28348344 | A | G | 0.00663154 | 0.00134803 | 8.68E-07 | 24.2007972 |
| rs7274597 | 20 | 34094549 | T | C | -0.0130623 | 0.00271678 | 1.53E-06 | 23.11694102 |
| rs73897246 | 20 | 15051571 | A | C | 0.00806784 | 0.00167751 | 1.51E-06 | 23.13046257 |
| rs61084367 | 21 | 42656370 | C | A | -0.00938119 | 0.00205261 | 4.87E-06 | 20.88829456 |

_*Delete SNP "rs2012697" and "rs1421085" as they are related to BMI._

_*Removing the SNP "rs2393986" for being palindromic with intermediate allele frequencies_

**Supplementary Table 5 Genetic instruments of Other type of coffee for Two-sample MR analysis**

| **SNP** | **chr** | **pos** | **effect_allele** | **other_allele** | **beta** | **se** | **pval** | **F.statistics** |
| --- | --- | --- | --- | --- | --- | --- | --- | --- |
| rs73173688 | 3 | 171172995 | A | G | 0.00493521 | 0.00106031 | 3.25E-06 | 21.66434483 |
| rs117539765 | 6 | 155425913 | A | G | -0.00434804 | 0.000948942 | 4.61E-06 | 20.99460576 |
| rs75994344 | 9 | 130620452 | A | G | 0.0081536 | 0.00164207 | 6.86E-07 | 24.65559403 |
| rs4899463 | 14 | 73914353 | G | C | 0.00828433 | 0.00171936 | 1.45E-06 | 23.21566578 |
| rs2074276 | 17 | 18863105 | T | C | -0.00171442 | 0.000343883 | 6.19E-07 | 24.85495809 |
| rs181147464 | 19 | 23375762 | C | T | 0.00327407 | 0.000693512 | 2.35E-06 | 22.28783893 |

**Supplementary Table 6 Genetic instruments of coffee intake for Two-sample MR analysis.**

| **SNP** | **chr** | **pos** | **effect_allele** | **other_allele** | **beta** | **se** | **pval** | **F.statistics** |
| --- | --- | --- | --- | --- | --- | --- | --- | --- |
| rs516636 | 1 | 177855517 | A | C | 0.0116767 | 0.00198419 | 4.00E-09 | 34.6316937 |
| rs4615895 | 1 | 96274668 | A | G | 0.0122025 | 0.00184972 | 4.20E-11 | 43.51967545 |
| rs13387939 | 2 | 637498 | A | C | 0.0165558 | 0.0021386 | 9.80E-15 | 59.92957774 |
| rs780093 | 2 | 27742603 | C | T | 0.0132935 | 0.00165695 | 1.00E-15 | 64.36649682 |
| rs12989746 | 2 | 49368391 | T | G | 0.01035 | 0.00186429 | 2.80E-08 | 30.8214992 |
| rs1527961 | 2 | 62780440 | C | T | -0.0133431 | 0.00236585 | 1.70E-08 | 31.80819827 |
| rs2597805 | 4 | 17424930 | T | C | 0.00985502 | 0.00175623 | 2.00E-08 | 31.48851914 |
| rs2189234 | 4 | 106075498 | G | T | 0.00998689 | 0.00166052 | 1.80E-09 | 36.17198243 |
| rs13163336 | 5 | 87943710 | A | C | 0.0149472 | 0.00221005 | 1.30E-11 | 45.74203614 |
| rs12514566 | 5 | 7391462 | A | G | -0.0113972 | 0.00170562 | 2.40E-11 | 44.65106059 |
| rs2465037 | 6 | 51179260 | A | C | -0.0106317 | 0.00170743 | 4.80E-10 | 38.77212562 |
| rs1338549 | 6 | 98312143 | G | T | -0.00945121 | 0.0016218 | 5.60E-09 | 33.96098155 |
| rs9398171 | 6 | 108983527 | T | C | 0.0108577 | 0.00177995 | 1.10E-09 | 37.21003427 |
| rs73075167 | 7 | 17570479 | T | A | -0.0160639 | 0.00244429 | 5.00E-11 | 43.19132446 |
| rs7811609 | 7 | 32930597 | T | C | 0.00913864 | 0.00166468 | 4.00E-08 | 30.13711083 |
| rs1057868 | 7 | 75615006 | T | C | 0.0199509 | 0.00178517 | 5.40E-29 | 124.9009737 |
| rs4410790 | 7 | 17284577 | C | T | 0.039072 | 0.00167288 | 1.20E-120 | 545.5087317 |
| rs34060476 | 7 | 73037956 | G | A | 0.0184292 | 0.00237033 | 7.50E-15 | 60.44986565 |
| rs6469262 | 8 | 110443480 | C | T | -0.00915347 | 0.00162895 | 1.90E-08 | 31.57592028 |
| rs78267637 | 8 | 33790200 | G | C | -0.0254259 | 0.00431659 | 3.90E-09 | 34.69533367 |
| rs442355 | 8 | 109128653 | C | G | -0.0111372 | 0.00185374 | 1.90E-09 | 36.09561078 |
| rs10119174 | 9 | 27953724 | C | G | -0.00939786 | 0.00164159 | 1.00E-08 | 32.77393185 |
| rs117810762 | 10 | 135315795 | A | G | 0.0359086 | 0.00617871 | 6.20E-09 | 33.77546389 |
| rs61928609 | 12 | 11316437 | C | A | -0.0147305 | 0.00217536 | 1.30E-11 | 45.85352204 |
| rs2472297 | 15 | 75027880 | T | C | 0.0464708 | 0.00182733 | 1.10E-142 | 646.7349474 |
| rs117968677 | 15 | 75174251 | A | G | -0.0310299 | 0.00551601 | 1.90E-08 | 31.6454053 |
| rs8056750 | 16 | 70927078 | T | C | 0.0105333 | 0.00173692 | 1.30E-09 | 36.77640515 |
| rs1421085 | 16 | 53800954 | C | T | 0.0185426 | 0.00164436 | 1.70E-29 | 127.159102 |
| rs62064918 | 17 | 46155786 | T | C | -0.0103075 | 0.00187872 | 4.10E-08 | 30.10111407 |
| rs57918684 | 17 | 60150383 | A | G | 0.0128864 | 0.00223845 | 8.60E-09 | 33.14121451 |
| rs7224815 | 17 | 17845800 | T | A | -0.0108602 | 0.00164162 | 3.70E-11 | 43.76534713 |
| rs630194 | 18 | 40950954 | C | T | -0.0113533 | 0.00169853 | 2.30E-11 | 44.67841767 |
| rs1942965 | 18 | 55032486 | C | T | -0.00890339 | 0.00161917 | 3.80E-08 | 30.23610867 |
| rs476828 | 18 | 57852587 | C | T | 0.0173461 | 0.00189543 | 5.60E-20 | 83.75065094 |
| rs56113850 | 19 | 41353107 | C | T | 0.0126667 | 0.00163351 | 8.90E-15 | 60.12891602 |
| rs75347775 | 19 | 18495908 | A | G | 0.0104504 | 0.00187898 | 2.70E-08 | 30.93296233 |
| rs6063085 | 20 | 45840459 | C | A | 0.0104106 | 0.00166919 | 4.50E-10 | 38.8991375 |
| rs6062682 | 20 | 62891820 | T | C | 0.0103704 | 0.00163929 | 2.50E-10 | 40.02021679 |
| rs13054099 | 22 | 41215672 | C | T | -0.0107777 | 0.00183597 | 4.30E-09 | 34.46045604 |
| rs17842490 | 22 | 24870527 | G | A | -0.0451683 | 0.00680848 | 3.30E-11 | 44.01160013 |

_*Removing the SNP "rs10119174" for being palindromic with intermediate allele frequencies._

_*Removing the following SNPs for being related to BMI:rs13163336, rs13387939, rs1421085, rs476828 ,rs516636, rs9398171._

**Supplementary Table 7 MR analysis for coffee intake and OA in various sites.**

| **outcome** | **exposure** | **method** | **nsnp** | **pval** | **OR** | **Low_95%CI** | **up_95%CI** |
| --- | --- | --- | --- | --- | --- | --- | --- |
| Self-reported OA | Coffee intake | MR Egger | 33 | 0.001 | 1.067 | 1.029 | 1.106 |
| Self-reported OA | Coffee intake | Weighted median | 33 | 0.001 | 1.043 | 1.017 | 1.070 |
| Self-reported OA | Coffee intake | Inverse variance weighted | 33 | 0.006 | 1.029 | 1.008 | 1.050 |
| Self-reported OA | Coffee intake | Simple mode | 33 | 0.47 | 0.972 | 0.901 | 1.049 |
| Self-reported OA | Coffee intake | Weighted mode | 33 | 7.50E-06 | 1.061 | 1.038 | 1.084 |
| KOA | Coffee intake | MR Egger | 33 | 0.001 | 2.458 | 1.522 | 3.967 |
| KOA | Coffee intake | Weighted median | 33 | 1.90E-04 | 2.009 | 1.393 | 2.897 |
| KOA | Coffee intake | Inverse variance weighted | 33 | 4.59E-08 | 2.026 | 1.573 | 2.609 |
| KOA | Coffee intake | Simple mode | 33 | 0.242 | 1.575 | 0.747 | 3.323 |
| KOA | Coffee intake | Weighted mode | 33 | 2.14E-05 | 2.498 | 1.742 | 3.583 |
| HOA | Coffee intake | MR Egger | 33 | 0.078 | 1.942 | 0.951 | 3.962 |
| HOA | Coffee intake | Weighted median | 33 | 0.073 | 1.451 | 0.966 | 2.180 |
| HOA | Coffee intake | Inverse variance weighted | 33 | 0.011 | 1.628 | 1.121 | 2.366 |
| HOA | Coffee intake | Simple mode | 33 | 0.664 | 1.215 | 0.509 | 2.896 |
| HOA | Coffee intake | Weighted mode | 33 | 0.070 | 1.539 | 0.980 | 2.417 |
| KOA/HOA | Coffee intake | MR Egger | 33 | 0.001 | 2.057 | 1.381 | 3.064 |
| KOA/HOA | Coffee intake | Weighted median | 33 | 8.32E-06 | 1.819 | 1.398 | 2.367 |
| KOA/HOA | Coffee intake | Inverse variance weighted | 33 | 1.68E-08 | 1.825 | 1.481 | 2.250 |
| KOA/HOA | Coffee intake | Simple mode | 33 | 0.056 | 1.938 | 1.007 | 3.731 |

**Supplementary Table 8 MR analysis for Decaffeinated coffee and OA in various sites.**

| **outcome** | **exposure** | **method** | **nsnp** | **pval** | **OR** | **Low_95%CI** | **up_95%CI** |
| --- | --- | --- | --- | --- | --- | --- | --- |
| Self-reported OA | Coffee intake | MR Egger | 16 | 0.457 | 0.939 | 0.800 | 1.103 |
| Self-reported OA | Coffee intake | Weighted median | 16 | 0.307 | 1.040 | 0.965 | 1.121 |
| Self-reported OA | Coffee intake | Inverse variance weighted | 16 | 0.102 | 1.044 | 0.991 | 1.100 |
| KOA | Coffee intake | MR Egger | 17 | 0.376 | 0.345 | 0.035 | 3.394 |
| KOA | Coffee intake | Weighted median | 17 | 0.185 | 2.238 | 0.680 | 7.369 |
| KOA | Coffee intake | Inverse variance weighted | 17 | 0.018 | 2.674 | 1.180 | 6.063 |
| HOA | Coffee intake | MR Egger | 17 | 0.002 | 0.003 | 0.000 | 0.062 |
| HOA | Coffee intake | Weighted median | 17 | 0.083 | 0.241 | 0.048 | 1.203 |
| HOA | Coffee intake | Inverse variance weighted | 17 | 0.097 | 0.338 | 0.094 | 1.219 |
| KOA/HOA | Coffee intake | MR Egger | 17 | 0.025 | 0.092 | 0.014 | 0.602 |
| KOA/HOA | Coffee intake | Weighted median | 17 | 0.894 | 0.934 | 0.340 | 2.562 |
| KOA/HOA | Coffee intake | Inverse variance weighted | 17 | 0.377 | 1.403 | 0.662 | 2.975 |

**Supplementary Table 9 MR analysis for Ground coffee and OA in various sites.**

| **outcome** | **exposure** | **method** | **nsnp** | **pval** | **OR** | **Low_95%CI** | **up_95%CI** |
| --- | --- | --- | --- | --- | --- | --- | --- |
| Self-reported OA | Coffee intake | MR Egger | 15 | 0.006 | 1.823 | 1.271 | 2.615 |
| Self-reported OA | Coffee intake | Weighted median | 15 | 0.206 | 0.959 | 0.899 | 1.023 |
| Self-reported OA | Coffee intake | Inverse variance weighted | 15 | 0.926 | 0.996 | 0.914 | 1.085 |
| KOA | Coffee intake | MR Egger | 17 | 0.001 | 5.27E+05 | 715.199 | 3.88E+08 |
| KOA | Coffee intake | Weighted median | 17 | 0.354 | 1.749 | 0.536 | 5.714 |
| KOA | Coffee intake | Inverse variance weighted | 17 | 0.796 | 1.229 | 0.257 | 5.888 |
| HOA | Coffee intake | MR Egger | 17 | 0.500 | 11.194 | 0.012 | 1.05E+04 |
| HOA | Coffee intake | Weighted median | 17 | 0.098 | 2.953 | 0.819 | 10.650 |
| HOA | Coffee intake | Inverse variance weighted | 17 | 0.375 | 1.690 | 0.531 | 5.385 |
| KOA/HOA | Coffee intake | MR Egger | 17 | 0.012 | 5.26E+03 | 14.226 | 1.94E+06 |
| KOA/HOA | Coffee intake | Weighted median | 17 | 0.009 | 3.456 | 1.366 | 8.742 |
| KOA/HOA | Coffee intake | Inverse variance weighted | 17 | 0.515 | 1.495 | 0.445 | 5.025 |

**Supplementary Table 10 MR analysis for Instant coffee and OA in various sites.**

| **outcome** | **exposure** | **method** | **nsnp** | **pval** | **OR** | **Low_95%CI** | **up_95%CI** |
| --- | --- | --- | --- | --- | --- | --- | --- |
| Self-reported OA | Coffee intake | MR Egger | 29 | 0.560 | 1.052 | 0.889 | 1.246 |
| Self-reported OA | Coffee intake | Weighted median | 29 | 0.877 | 1.004 | 0.955 | 1.055 |
| Self-reported OA | Coffee intake | Inverse variance weighted | 29 | 0.903 | 0.997 | 0.944 | 1.052 |
| KOA | Coffee intake | MR Egger | 32 | 0.431 | 3.207 | 0.183 | 56.135 |
| KOA | Coffee intake | Weighted median | 32 | 0.793 | 0.905 | 0.430 | 1.904 |
| KOA | Coffee intake | Inverse variance weighted | 32 | 0.911 | 1.052 | 0.434 | 2.546 |
| HOA | Coffee intake | MR Egger | 32 | 0.944 | 0.892 | 0.037 | 21.212 |
| HOA | Coffee intake | Weighted median | 32 | 0.750 | 0.856 | 0.328 | 2.233 |
| HOA | Coffee intake | Inverse variance weighted | 32 | 0.380 | 0.646 | 0.244 | 1.714 |
| KOA/HOA | Coffee intake | MR Egger | 32 | 0.662 | 1.747 | 0.147 | 20.717 |
| KOA/HOA | Coffee intake | Weighted median | 32 | 0.822 | 1.075 | 0.574 | 2.015 |
| KOA/HOA | Coffee intake | Inverse variance weighted | 32 | 0.747 | 0.882 | 0.412 | 1.889 |

**Supplementary Table 11 MR analysis for Instant coffee and OA in various sites.**

| **outcome** | **exposure** | **method** | **nsnp** | **pval** | **OR** | **Low_95%CI** | **up_95%CI** |
| --- | --- | --- | --- | --- | --- | --- | --- |
| Self-reported OA | Coffee intake | MR Egger | 6 | 0.049 | 0.462 | 0.268 | 0.795 |
| Self-reported OA | Coffee intake | Weighted median | 6 | 0.082 | 0.706 | 0.476 | 1.045 |
| Self-reported OA | Coffee intake | Inverse variance weighted | 6 | 0.322 | 0.818 | 0.550 | 1.217 |
| KOA | Coffee intake | MR Egger | 6 | 0.139 | 4.81E-05 | 1.24E-09 | 1.870 |
| KOA | Coffee intake | Weighted median | 6 | 0.268 | 0.024 | 3.43E-05 | 17.440 |
| KOA | Coffee intake | Inverse variance weighted | 6 | 0.203 | 0.021 | 5.20E-05 | 8.168 |
| HOA | Coffee intake | MR Egger | 6 | 0.444 | 0.005 | 2.61E-08 | 997.968 |
| HOA | Coffee intake | Weighted median | 6 | 0.872 | 1.800 | 0.001 | 2256.387 |
| HOA | Coffee intake | Inverse variance weighted | 6 | 0.493 | 0.122 | 2.99E-04 | 49.849 |
| KOA/HOA | Coffee intake | MR Egger | 6 | 0.216 | 3.60E-04 | 9.00E-09 | 14.412 |
| KOA/HOA | Coffee intake | Weighted median | 6 | 0.504 | 0.174 | 0.001 | 29.354 |
| KOA/HOA | Coffee intake | Inverse variance weighted | 6 | 0.339 | 0.061 | 1.97E-04 | 18.836 |

**
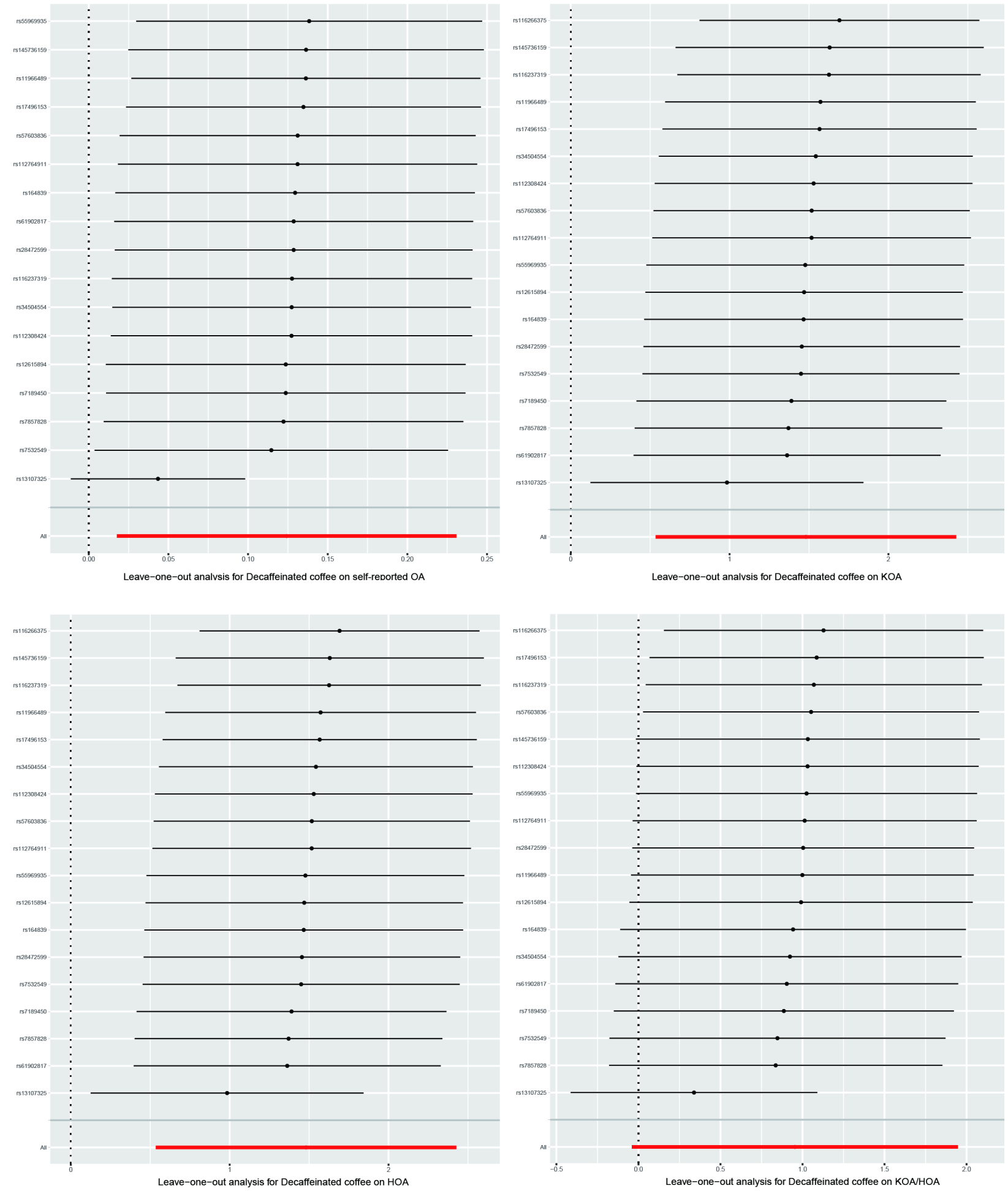
**

**Supplementary figure 1 MR Leave-one-out sensitivity analysis for Decaffeinated coffee and OA in various sites.**

**
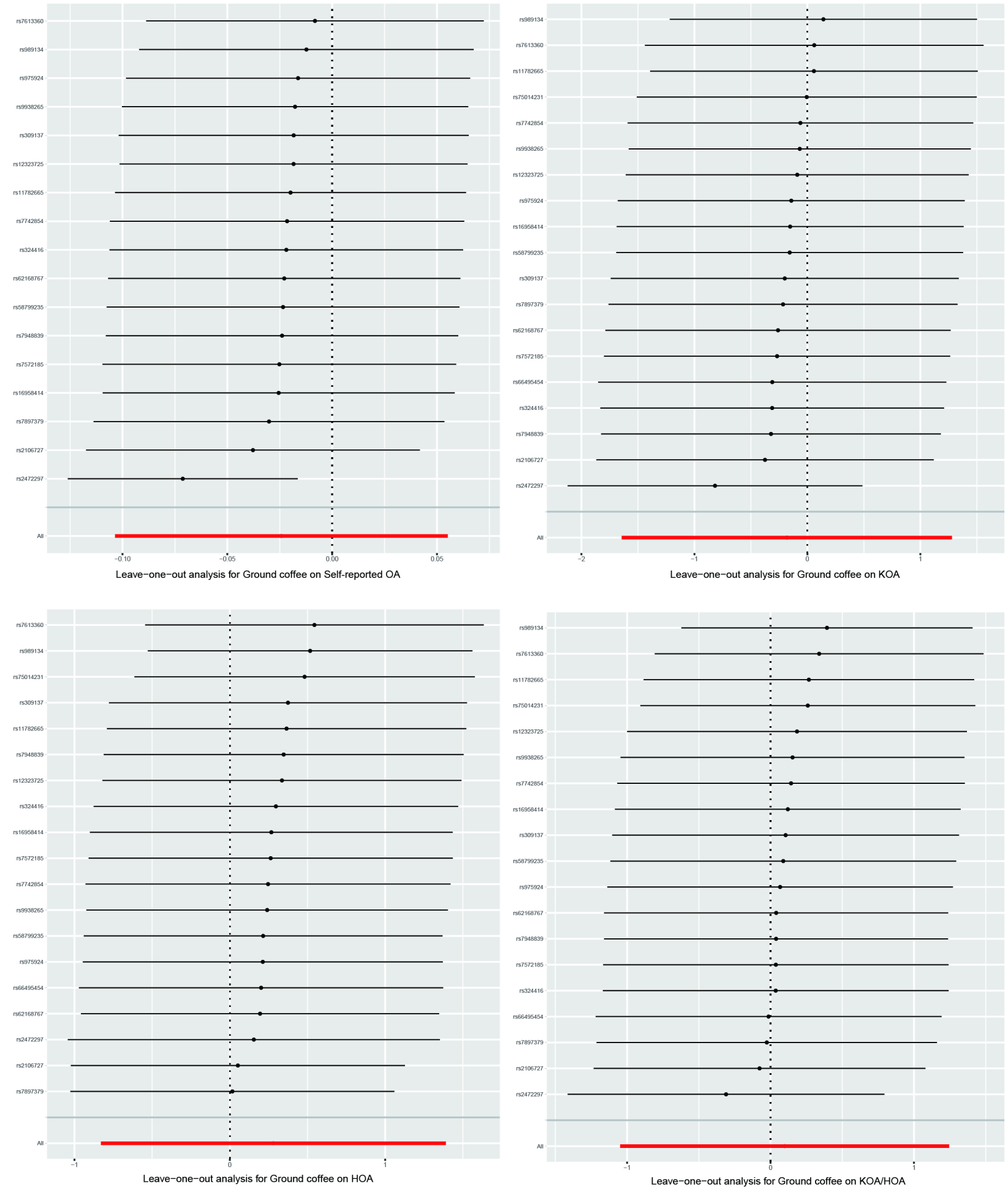
**

**Supplementary figure 2 MR Leave-one-out sensitivity analysis for Ground coffee and OA in various sites.**

**
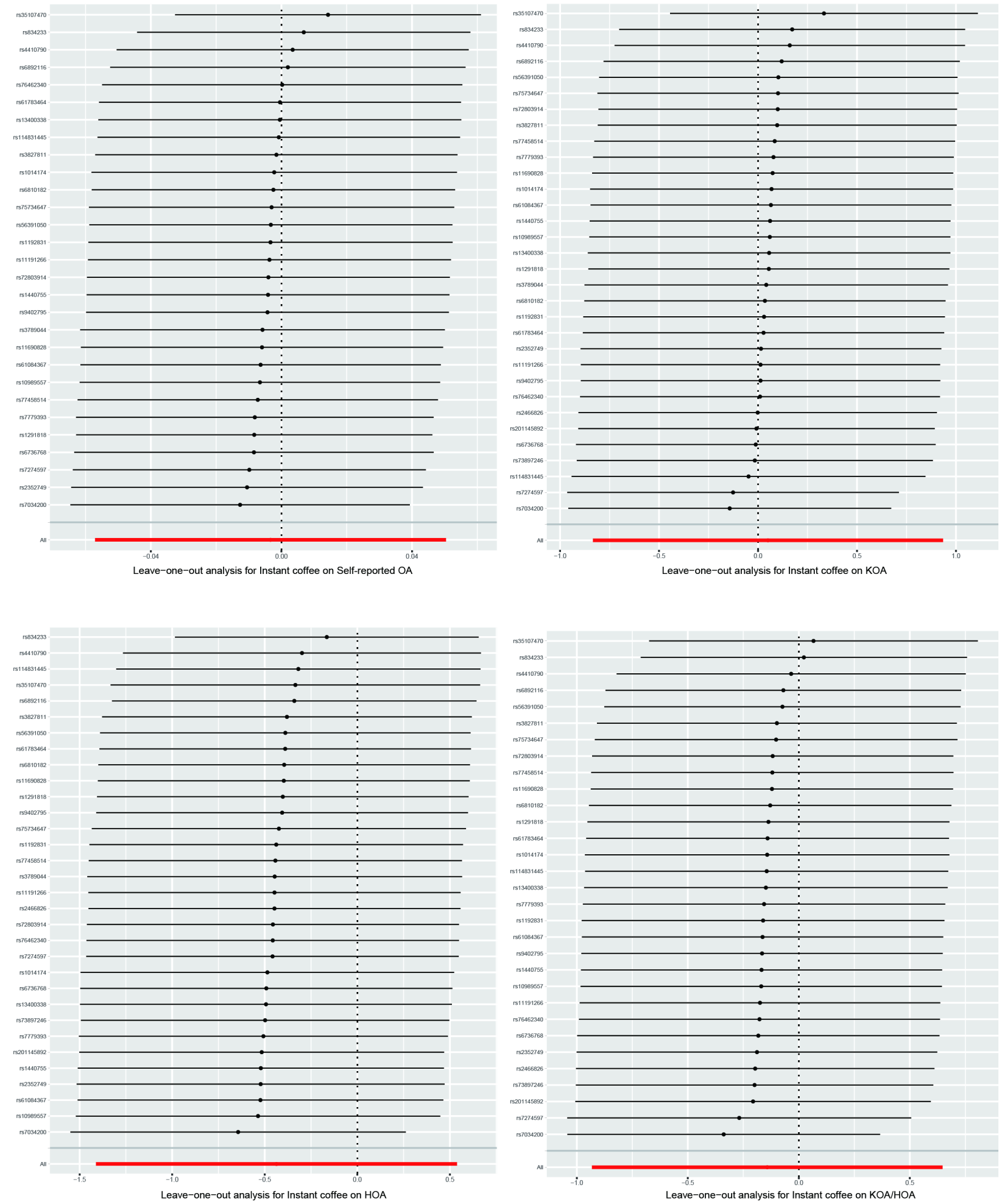
**

**Supplementary figure 3 MR Leave-one-out sensitivity analysis for Instant coffee and OA in various sites.**

**
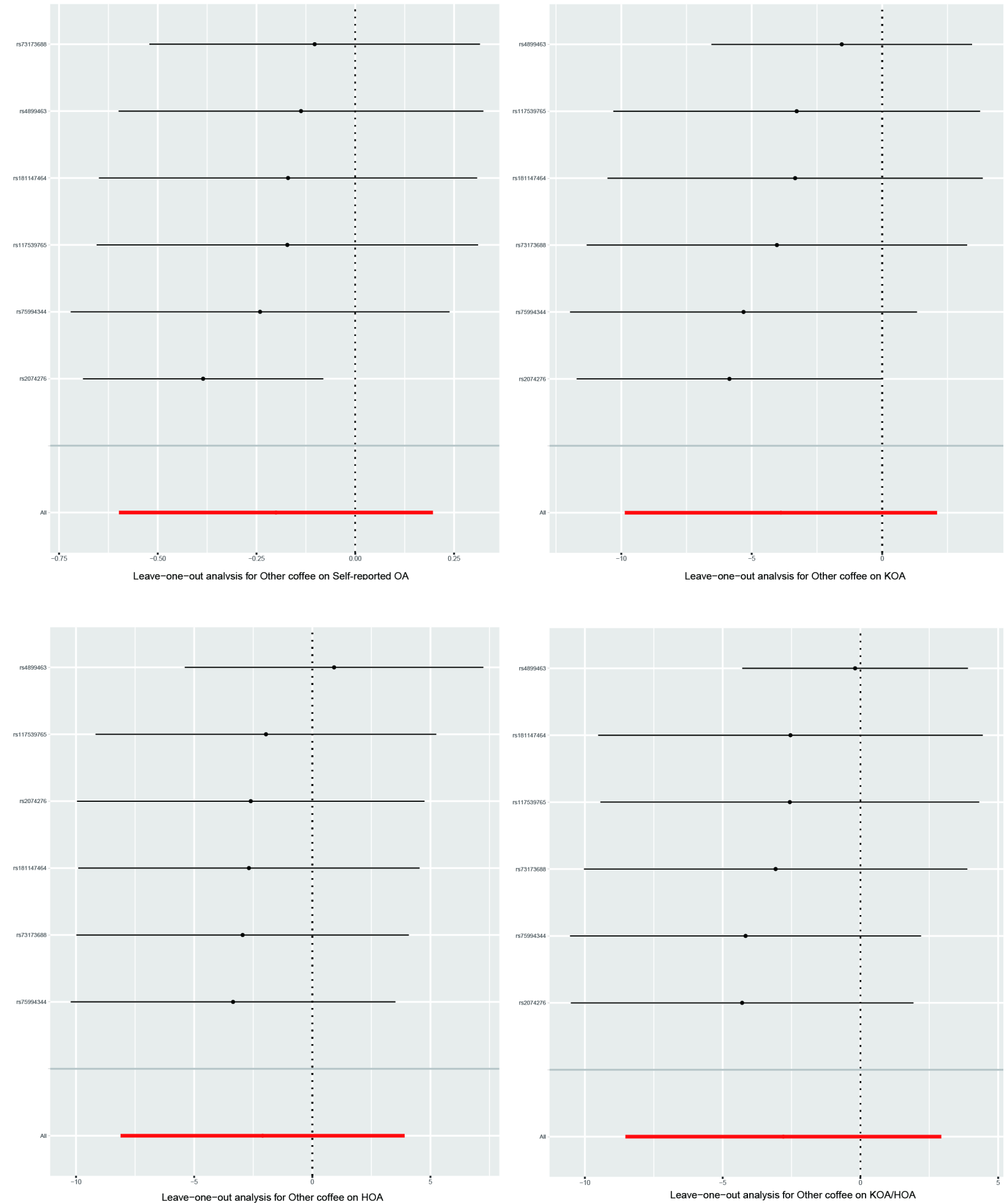
**

**Supplementary figure 4 MR Leave-one-out sensitivity analysis for Other coffee and OA in various sites.**
